# Inhibition of the Protein Arginine Methyltransferase PRMT5 in High-Risk Multiple Myeloma as a Novel Treatment Approach

**DOI:** 10.3389/fcell.2022.879057

**Published:** 2022-06-08

**Authors:** Philip Vlummens, Stefaan Verhulst, Kim De Veirman, Anke Maes, Eline Menu, Jérome Moreaux, Hugues De Boussac, Nicolas Robert, Elke De Bruyne, Dirk Hose, Fritz Offner, Karin Vanderkerken, Ken Maes

**Affiliations:** ^1^ Department of Hematology and Immunology, Vrije Universiteit Brussel (VUB), Brussels, Belgium; ^2^ Department of Clinical Hematology, Ghent University Hospital, Ghent, Belgium; ^3^ Liver Cell Biology Lab, Vrije Universiteit Brussel (VUB), Brussels, Belgium; ^4^ CHU Montpellier, Laboratory for Monitoring Innovative Therapies, Department of Biological Hematology, Montpellier, France; ^5^ Department of Biological Hematology, CHU Montpellier, Montpellier, France; ^6^ Institut Universitaire de France, IUF, Paris, France; ^7^ Center for Medical Genetics, Vrije Universiteit Brussel (VUB), Universitair Ziekenhuis Brussel (UZ), Brussels, Belgium

**Keywords:** myeloma, PRMT5, DNA repair, RNA splicing, epigenetics

## Abstract

Multiple myeloma (MM) is an incurable clonal plasma cell malignancy. Subsets of patients have high-risk features linked with dismal outcome. Therefore, the need for effective therapeutic options remains high. Here, we used bio-informatic tools to identify novel targets involved in DNA repair and epigenetics and which are associated with high-risk myeloma. The prognostic significance of the target genes was analyzed using publicly available gene expression data of MM patients (TT2/3 and HM cohorts). Hence, protein arginine methyltransferase 5 (PRMT5) was identified as a promising target. Druggability was assessed in OPM2, JJN3, AMO1 and XG7 human myeloma cell lines using the PRMT5-inhibitor EPZ015938. EPZ015938 strongly reduced the total symmetric-dimethyl arginine levels in all cell lines and lead to decreased cellular growth, supported by cell line dependent changes in cell cycle distribution. At later time points, apoptosis occurred, as evidenced by increased AnnexinV-positivity and cleavage of PARP and caspases. Transcriptome analysis revealed a role for PRMT5 in regulating alternative splicing, nonsense-mediated decay, DNA repair and PI3K/mTOR-signaling, irrespective of the cell line type. PRMT5 inhibition reduced the expression of upstream DNA repair kinases ATM and ATR, which may in part explain our observation that EPZ015938 and the DNA-alkylating agent, melphalan, have combinatory effects. Of interest, using a low-dose of mTOR-inhibitor, we observed that cell viability was partially rescued from the effects of EPZ015938, indicating a role for mTOR-related pathways in the anti-myeloma activity of EPZ015938. Moreover, PRMT5 was shown to be involved in splicing regulation of MMSET and SLAMF7, known genes of importance in MM disease. As such, we broaden the understanding of the exact role of PRMT5 in MM disease and further underline its use as a possible therapeutic target.

## Introduction

Multiple myeloma (MM) is a clonal B-cell malignancy characterized by the proliferation of malignant plasma cells in the bone marrow. The disease is characterized by marked complexity of genomic defects, which is believed to trigger the progression from monoclonal gammopathy of undetermined significance (MGUS), a pre-malignant stage, towards florid MM disease (Joseph et al., 2017; Binder et al., 2019). Moreover, high-risk genomic defects can already be seen in clonal plasma cells in the MGUS stage, thus leading to high-risk disease even at the MGUS and smoldering MM (SMM) stage (Keuhl and Bersagel, 2012; Hajek et al., 2013). In recent years, emerging evidence has shown that epigenetic alterations are involved in MM pathogenesis and disease progression. Moreover, targeting of several epigenetic modifiers such as DNA methyl transferases and histone deacetylases has shown to be able to exhibit anti-MM effects (Caprio et al., 2020). As relapse in high-risk patients cannot be avoided with current treatment options, despite availability of novel agents and monoclonal antibodies, additional treatment strategies that focus on novel targets are needed. Therefore, a deeper understanding of MM disease biology focusing on epigenetic targets is of high value because this can lead to novel therapeutic options.

One important mechanism of epigenetic and post-translational modifications in cancer cells is arginine methylation (Gulla et al., 2018). As such, it was identified that protein methyltransferases (PRMT) play a role in the modulation of gene transcription and protein function and play critical roles in regulatory pathways leading to cancer development and therapy resistance (Kim and Ronai, 2020). One of these is PRMT5, a type II PRMT enzyme which has been shown to play a role in tumour development and progression in solid cancer (Tan et al., 2020; Banasavadi-Siddegowda, 2017). PRMT5 is involved in lymphomagenesis through the inhibition of p53-dependant tumour suppression in response to oncogenic events (Li et al., 2015). It was also shown that MYC directly upregulates the transcription of the core small nuclear ribonucleoprotein particle assembly genes, including PRMT5, regulating splicing machinery with an essential role in lymphomagenesis (Koh et al., 2015). Moreover, its role in MM pathogenesis has been reported by Gulla et al. (Gulla et al., 2018). They showed that PRMT5 has a prognostic role in MM patients and that it is implicated in NF-kB signaling in MM cells.

Here, we report the independent identification of PRMT5 using large public datasets and further elucidate the role in MM disease, focusing on pathophysiology and therapeutic implications. In detail, we implicate PRMT5 as an important driving force in MM cell signaling, showing its involvement in DNA damage repair, mTOR signaling and mRNA splicing.

## Materials and Methods

### Bioinformatical Analysis and PRMT5 Identification

Publicly available data sets were used to evaluate the prognostic significance of 457 selected genes, known to be involved in epigenetic regulation. The University of Arkansas for Medical Sciences (UAMS) cohort consists of 345 MM patients treated with thalidomide versus placebo combined with 4 cycles of chemotherapy (Total Therapy 2/3 protocol or TT2/3). Data can be accessed through the Gene Expression Omnibus, accession number GSE2658. (Zhan et al., 2007; Barlogie et al., 2006). Normalised gene expression data was used for further analysis and coupled with patient survival data when available. The second dataset used for identification was the Heidelberg-Montpellier cohort, an independent MM patient cohort consisting of 206 patients for whom complete survival data was available. This cohort also includes 7 bone marrow plasma cell (BMPC) samples from healthy donors, which were not used in our analysis. These data are publicly available through ArrayExpress database (E-MTAB-372) (Hose et al., 2009; Hose et al., 2011). Subsequent validation of withheld candidate genes was performed in 2 independent cohorts. The GMMG-HD4/HOVON-65 cohort consists of gene expression data of 320 newly diagnosed MM patients for which survival data is available (Broyl et al., 2010). The Mulligan patient cohort, consisting of 264 bone marrow aspirate samples of patients with relapsed multiple myeloma, treated with dexamethasone versus bortezomib, was used for validation (GEO accession number GSE9782) (Mulligan et al., 2007; Weinhold et al., 2016). PRMT5 was selected out of the prognostic gene list because of the potential druggability and unexplored mode of action. Validation of PRMT5 as a candidate gene was performed using the Relating Clinical Outcomes in MM to Personal Assessment of Genetic Profile (CoMMpass) trial release IA14, launched by the MMRF. Normalized FPKM gene expression values, generated using RNA-sequencing, were downloaded alongside clinical data through the MMRF research portal (https://research.themmrf.org). Collected baseline data included the presence of chromosomal abnormalities and patient survival data. Gene expression levels were correlated with patient survival data using the MaxStat R package as previously described, as such analysing the prognostic value of the genes of interest.

### Cell Lines and Cell Culture

Human MM cell lines (HMCLs) OPM2, JJN3 and AMO1 obtained from ATCC (Molsheim, France). The XG7 HMCL was kindly provided by Jérôme Moreaux (University of Montpellier) (Moreaux et al., 2011; Vikova et al., 2019). JJN3 have a del17p with deletion of one p53 locus whereas OPM2 cells harbour a p53 mutation (Moreaux et al., 2011). Cells were cultured in RMPI1640 medium (Lonza, Basel, Switzerland) supplemented with 10% fetal bovine serum (FBS) (Hycone, Logan, United States), 2 mM L-glutamin and 1% penicillin/streptomycin (Thermo Fisher Scientific, Waltham, United States) at 37°C in a 5% CO_2_ enriched atmosphere. As the XG7 cell line is interleukin 6 (IL6) dependent, recombinant IL6 (R&D Systems, Minneapolis, United States) was added at a concentration of 2 ng/ml for this cell line specifically. All HMCLs were regularly tested for *mycoplasma* contamination and checked for authenticity by STR profiling.

### Treatment Schedules

PRMT5 inhibition was performed by culturing HMCLs (0.1 × 10^6 cells/ml) with or without EPZ015938 (Selleckchem, Munich, Germany) at a concentration of 5 or 10 µM. DMSO at a concentration of 1 % was added to the control sample. Refreshing the cells: cells were gently harvested, counted and replated at the initial cellular concentration (0.1 × 10^6 cells/ml) on day 3 and 6 by in fresh mediumto which either DMSO or EPZ015937 was added. Cells were collected at indicated timepoints for subsequent analysis. Combination experiments were performed with melphalan (Selleckchem, Munich, Germany) and the mTOR-inhibitor KU-0063794 (Selleckchem, Munich, Germany). For combination experiments with melphalan, HMCLs were exposed to EPZ015938 for 3 days (OPM2, AMO1, JJN3) or 1 day (XG7) prior to adding melphalan at different concentrations (5 or 10 µM) for two additional days. Combination experiments with KU-0063794 were performed by exposing HMCLs to EPZ015938, KU-0063794 or the combination for 6 days (OPM2, AMO1, JJN3) or 4 days (XG-7). Concentration of the mTOR-inhibitor (0.5 up to 10 µM) and time of analysis (day 4 or 6) was determined according to basal HMCL susceptibility to the drug.

### Treatment of Primary MM Cells

Bone marrow of MM patients (*n* = 7) was obtained at the university hospital of Montpellier after patients’ written informed consent in accordance with the Declaration of Helsinki and agreement of the Montpellier University Hospital Centre for Biological Resources (DC-2008-417). Mononuclear cells (MMC) were treated with or without EPZ015938 (2 μM) and MMC cytotoxicity was evaluated using an anti-CD138-phycoerythrin monoclonal antibody (Immunotech, Marseille, France) as described previously (De Boussac H et al., 2020).

### Growth Assessment, Apoptosis Assay and Cell Cycle Analysis

Cell growth was assessed on day 3 by manual trypan blue counting. Viability and apoptosis were assessed at predefined timepoints. Briefly, cells were analysed using AnnexinV/FITC-staining (BD Biosciences, Belgium) and 7-AAD staining (BD Biosciences) by flow cytometry on a BD FACSCanto Clinical Flow Cytometry System using the manufacturer’s instructions. Cell cycle analysis was performed on day 3 or 4 of treatment according to cell line type. Cells were stained for 5 min with a PI solution containing 1 mg/ml sodium nitrate (Merck, Darmstadt, Germany), 0.1% Triton-X (Merck), 100 μg/ml RNase A (Boehringer, Ingelheim, Germany) and 50 μg/ml PI (Sigma-Aldrich, Overijse, Belgium). Analysis was subsequently performed using flow cytometry (BD FACSCanto Clinical Flow Cytometry System).

### Western Blot Analysis

Cells were harvested and lysed prior to western blotting as previously described (De Bruyne et al., 2010). Analysis was performed using chemiluminescent detection using Li-Cor Odyssey Fc (Li-Cor Biosciences, Lincoln, United States). Antibodies used were targeted against PRMT5 (#79998), SDMA (#13222), Beta-actin (#4967), caspase-9 (#9502), caspase-3 (#9662), PARP (#9542), p-Ser15-p53 (#9286), p53 (#9282), p27 (#3688), p21 (#2974), ATM (#2873), p-ATM (#4526), FANCA (#14657S), ATR (#2792), p-ATR (#2853), tubulin (#2144), p-AMPK (#2535), AMPK (#5831), p-4EBP1 (#2855), 4EBP1 (#9644), MMSET (#65127), HELLS (#7998) and SLAM7 (#98611). HRP-linked anti-mouse IgG (#7076S) and anti-rabbit IgG (7074S) were used for primary antibody detection. Antibodies were purchased from Cell Signaling Technology (Leiden, the Netherlands). Densitometric analysis of western blot data was perfomed using Image Studio (Li-Cor Biosciences).

### Immunofluorescent Staining and Microscopy

Cells were plated as described above. After 3 days, cytospins were made and stored at -20°C. Cytospins were subsequently stained for gamma-H2AX as previously described (Maes et al., 2014). Immunofluorescence was observed using a Nikon Eclipse 90i with a ×40 objective magnification and ×10 ocular magnification. Pictures were taken using a Nikon DS-Ri1.

### RNA Sequencing and Bioinformatical Analysis

Selected HMCLs were cultured for 3 days with either EPZ015938 or placebo (DMSO). RNA was extracted as previously described (Vikova et al., 2019). Sample quality was checked by calculating the RNA integrity number (RIN value). RNA-seq library preparation was done with 150 ng of input RNA using the Illumina TrueSeq Stranded mRNA Library Prep Kit (Illumina, Cambridge, UK). Paired-end RNA-seq was performed on an Illumina sequencing instrument (Helexio, Clermond-Ferrand, France). Read pairs were mapped to the human reference genome (version GRCh38) using the STAR alignment algorithm. Differential expression analysis was performed using the R/BioconductorDESeq2 package with *p*-value adjustment for multiple comparisons using the default option (Love et al., 2014). Genes were considered differentially expressed when having a *p*-value ≤ 0.05 and a fold change of 1.5 in either direction. Intron retention analysis was performed using the R/Bioconductor IRFinder package according to the package vignette (Middleton et al., 2017). Geneset annotation and pathway enrichment analysis were performed using the R/Bioconductor ReactomePA package (Yu and He, 2016). PCA analysis was performed using R/Bioconductor Rtsne package (van der Maaten and Hinton, 2008).

### qPCR Analysis

Total RNA was extracted using the Nucleospin RNA plus kit (Macherey-Nagel, Düren, Germany), including gDNA removal. Reverse transcription was performed using the Verso cDNA synthesis kit (ThermoFisher Scientific, Gent, Belgium), both according to manufacturer’s instructions. Quantitative real-time PCR was performed as previously described (Oudaert et al., 2022). Primers were purchased from IDT (Leuven, Belgium). Primers were designed to target the intronic region of the gene of interest. Sequences are shown in Supplementary Figure S13.

### Statistical analysis

Prognostic significance of gene expression levels was calculated as indicated above. Overall survival (OS) is defined as the time from trial inclusion until death from any cause or until the time point the patient was last known to be alive. In the latter case patients were censored. Progression free survival (PFS) delineates the time from treatment initiation until relapse or death from any cause. Statistical analysis was performed using the IBM SPSS software package, v. 26 (Chicago, IL, United States). Survival curve estimates were generated using the Kaplan-Meier method and statistical significance was calculated using the log-rank test. Statistical significance was determined using a Mann-Whitney (two conditions) or one –way ANOVA with Bonferroni’s multiple comparison test for selected pairs paired T-test (multiple conditions). Correlation coefficients of RNAseq data were calculated by using the Pearson method. Reported *p*-values are 2-sided and a conventional significance level of 5% was used. Combination index (CI) values were calculated for the EPZ015938—melphalan combination experiments by the Chou and Thalalay method using CompuSyn 1.0 software.

## Results

### Bioinformatical Analysis Identifies PRMT5 as a Gene Linked to Poor Prognosis Gene in MM

A total of 457 candidate genes, involved in epigenetic regulation and DNA-repair, were identified for further analysis (Supplementary Table S1). MaxStat analysis was performed in both the TT2/3 and HM patient cohorts thus generating a list of genes being prognostic in both cohorts internally (Hothorn and Lausen, 2003). Using this approach, a common set of 45 poor and 17 good prognostic genes were identified (Supplementary Table S1). To exclude the effect of known prognostic drivers in MM, WHSC1 was excluded from this analysis (data not shown). Performing unsupervised hierarchical clustering of the TT2/3 and HM cohort using these common genes lead to a clear delineation of a high-risk MM population. A clear increase in hazard rate was seen in both cohorts; HR = 2.86 with *p* = 0.0017 in the HM and HR 2.22 with *p* = 0.0032 in the MMRF cohort (Supplementary Figure S1). Subsequent analysis of the HOVON and Mulligan cohort revealed similar OS results, as a HR = 2.5 with *p* < 0.0001 and HR = 2.04 with *p* = 0.0025 was seen respectively (Supplementary Figure S2). Lastly, our signature retained its prognostic value in an RNA-seq based cohort (MMRF), both at the level of PFS (HR = 2.13, *p*-value < 0.0001) and OS (HR = 2.92, *p* < 0.0001) (Supplementary Figure S3). From the 45 candidate genes, we chose PRMT5 for which the biological function is partially known, and clinical grade inhibitors are available for further study (Supplementary Figure S4).

### PRMT5 Inhibition in HMCLs Leads to Decreased Cellular Growth and Increased Apoptosis

**FIGURE 1:**
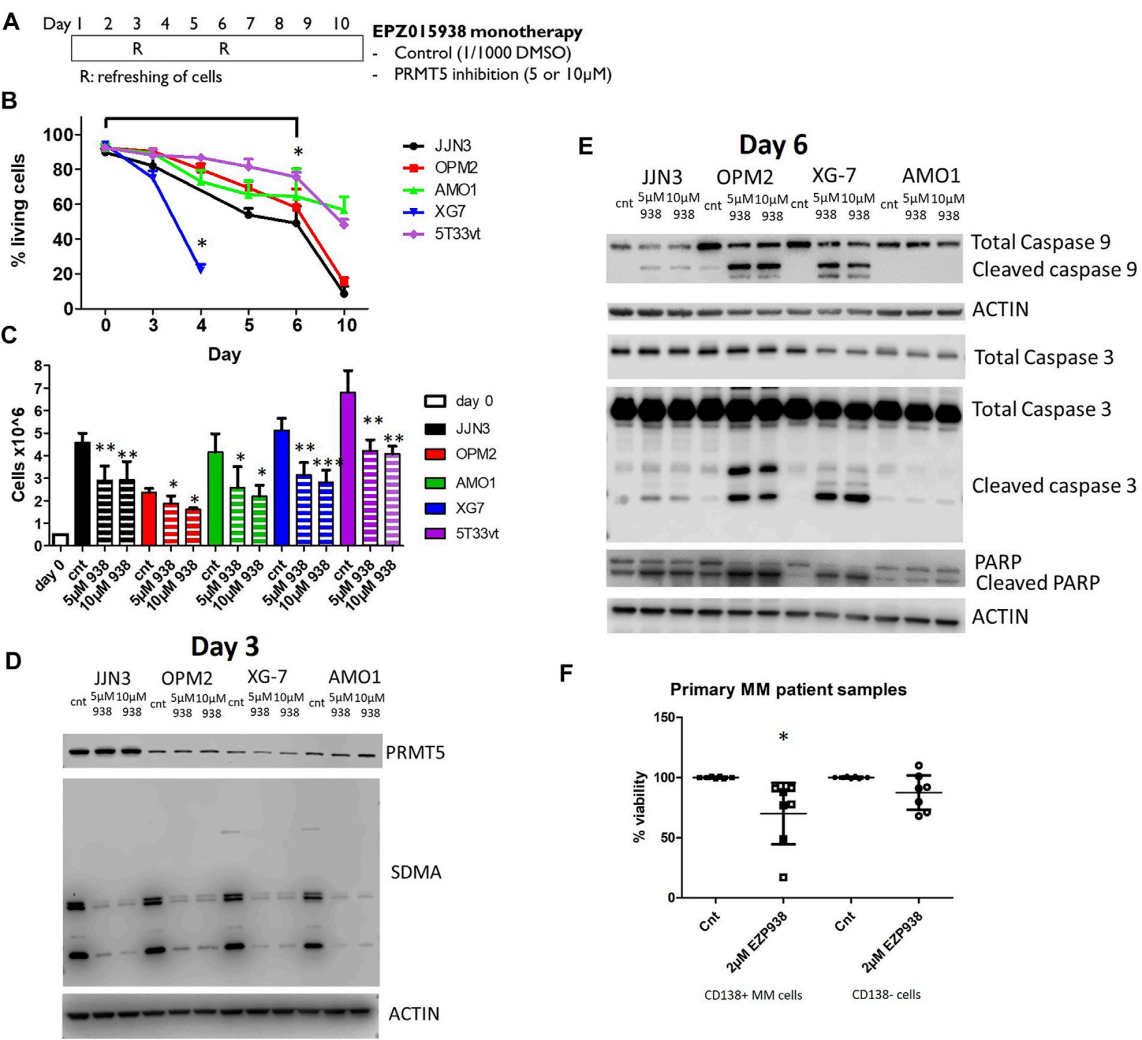
**(A)**: Schematic representation of EPZ01938 therapy in HMCLs. Cells were incubated with either compound or DMSO as placebo. Cells were refreshed at predetermined intervals. At timepoints R cells were gently harvested, counted and replated at the initial cellular concentration (0.1 × 10^6 cells/ml) on day 3 and 6 by in fresh medium to which either DMSO or EPZ015938 was added. **(B)**: Viability assay of HMCLs upon treatment with EPZ015938. Viability was assessed by trypane blue staining at depicted timepoints for the selected HMCLs. Treated samples were compared against control samples. Error bars depict mean values ± SD; * denotes *p* < 0.05 (*n* > 3) **(C)**: Cumulative cell counts of living cells for each HMCL when cultured with or without 5 and 10 µM EPZ015938 after 3 days of treatment. Treated samples were compared against control samples. Error bars depict mean values ± SD; */**/*** denotes *p* < 0.05, *p* < 0.01 and *p* < 0.001 respectively (*n* > 3) **(D)**: Western blot staining for PRMT5 and SDMA protein levels was performed after 3 days of EPZ015938 treatment on JJN3, OPM2, XG7 and AMO1 cells. Actin was used as a loading control. One experiment representative of 3 experiments performed is shown (*n* = 3). **(E)**: Western blot of pro-apoptotic proteins caspase 9, 3 and PARP after 3 days of treatment with EOZ015938. Actin was added as loading control. One representative experiment is shown (*n* = 3). **(F)**: Effect of PRMT5 inhibitor treatment on primary human CD138 + MM cells and CD138- microenvironment. Mononuclear cells from 7 MM patients were treated with the indicated concentration for 4 days, and the percentage of viable CD138 + plasma cells and CD138- cells were determined by flow cytometry. Results are expressed as the relative viability compared with control. * denotes *p* < 0.05 compared to control cells.

We inhibited PRMT5 function in 4 HMCLs using the EPZ015938 compound. EPZ015938 is an orally active and selective inhibitor of PRMT5 which is currently also being used in clinical trials (clinicaltrials.gov, accessed 04-Apr-22). Cells were exposed to different concentrations of EPZ015938 for up to 10 days according to the refreshment scheme (Figure 1A). A clear decrease (*p* < 0.05) in cell viability could be seen using Annexin-V/7AAD staining, with XG-7 cells being the most susceptible to EPZ015938 induced apoptosis with a significant decrease in cellular viability becoming apparent at day 4. In other HMCLs, a similar effect was observed after 6 days of treatment. AMO1 cells were shown to be the most resistant to effects of PRMT5 inhibition (Figure 1B). Moreover, significantly (*p* < 0.05) decreased cellular growth was observed in all cell lines from day 3 (Figure 1C). To further evaluate this observation, cell cycle analysis was performed. A cell line dependent effect on cell cycle could be seen (Supplementary Figure S5A). In both JJN3 and OPM2 cells, an increase in G2 was observed, while in AMO1 an increase in G1 could be seen. Both AMO1 and OPM2 cells showed decrease cell numbers in S-phase was seen alongside an increase of cells in the G2-phase. In XG-7 cells a significant increase in sub-G1 was observed, because of increased cell death already visible at this time point. On a protein level, an increase in both phosphorylated and unphosphorylated p53 levels were seen in all but JJN3 cells, which have a known bi-allelic deletion of the p53 gene. Only a slight increase could be seen in AMO1 cells. Effects on p21 and p27, G1-checkpoint cyclin-dependant kinase inhibitors involved in p53-mediated apoptosis differed between cell lines as a decrease in p21 levels was observed in JJN3 and OPM2 cells whereas an increase was observed in XG7 and AMO1 cells. p27 levels showed a relative increase after PRMT5 inhibition in AMO1 cells but not in the other HMCLs studied (Supplementary Figure S5B). We also evaluated the effect of EPZ015938 treatment on global symmetrical di-methylated arginine (SDMA) residues as well as PRMT5 protein levels to ensure inhibitor target function and exclude autoregulation of PRMT5. Western blotting of cell lysates showed a decrease in SDMA levels, suggestive of on target effects of EPZ015938, alongside unaltered PRMT5 levels (Figure 1D). The presence of apoptosis, in view of prior observations of the effect of PMRT5 inhibition on cellular survival, was also confirmed on the protein level as an activation of the pro-apoptotic proteins caspase 3, caspase 9 and PARP could be objectified (Figure 1E). The least activation could be seen in AMO1 cells, which corroborates with the observation of lowered sensitivity to EPZ015938. Treatment of primary human MM samples (n = 7, clinical data Supplementary Table S1) showed a similar variability in response upon exposure to EPZ015938 but a significant decrease in MM cell viability could also be observed (Figure 1F). Additionally, no significant effect was observed on the CD138- negative cellular fraction from the bone marrow microenvironment (Figure 1F).

### RNA-Sequencing Identifies PRMT5 as a Modulator of DNA Repair, mTOR Signaling and Alternative Splicing in MM

**FIGURE 2:**
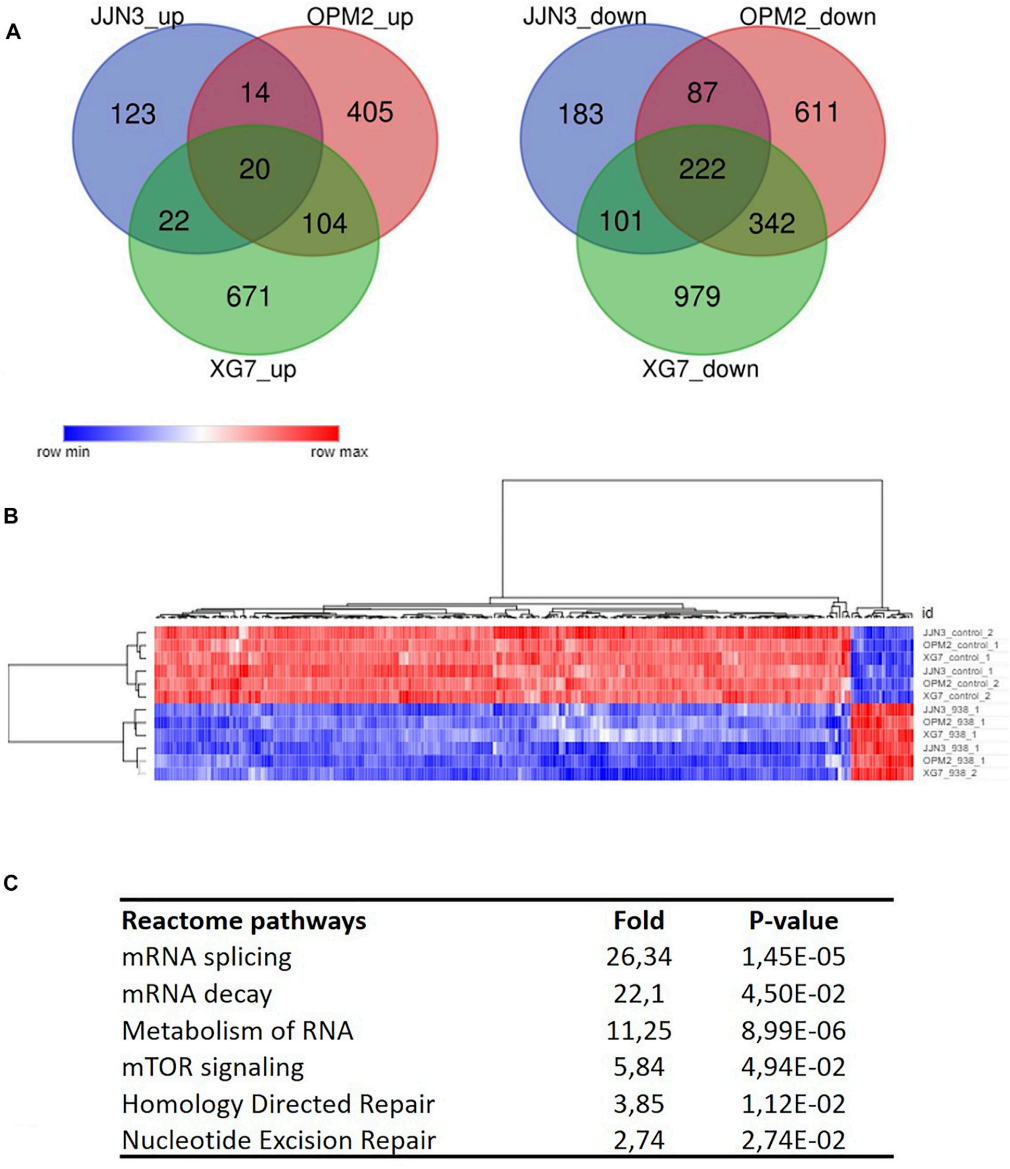
**(A)**: Deregulated genes after PRMT5 inhibition were identified using RNA-seq and analysed for JJN3, OPM2 and XG7 cells separately due to possible cell type specific events. Venn diagram analysis shows the presence of a specific set of common up- (left) or downregulated (right) genes per cell line type (*n* = 2 × 2 per cell type/treatment). **(B)**: Unsupervised clustering of analysed samples used for RNA-seq based on common deregulated genes (*n* = 242), clearly segregating according to treatment type. **(C)**: Overview of reactome pathway analysis output (selected pathways are shown) with fold changes and *p*-values per pathway, after multiple testing correction.

To molecularly assess the impact of PRTM5 inhibition, we performed RNA-seq on OPM2, JJN3 and XG7 cells with and without the addition of EPZ015938 (*n* = 2 for each condition). Primary analysis of sequencing data confirmed an influence of HMCL type on normalized counts (Supplementary Figure S5). DESeq2 analysis per cell line was used to identify differentially expressed genes (Supplementary Table S2). A common set of 20 up- and 222 downregulated genes could be identified alongside HMCL specific gene sets (Figures 2A,B; Supplementary Table S2). To identify common pathways where PRMT5 is involved, further analysis was performed on the common gene set only. Reactome gene set enrichment revealed that EPZ015938 treatment influenced mRNA splicing pathways, mTOR signalling and DNA repair mechanisms in all 3 HMCLs (Figure 2C). We subsequently selected these pathways for further functional investigation.

### PRMT5 Inhibition Modulates DNA Repair Mechanisms in MM and Leads to Increased Cell Death When Combined With Melphalan

**FIGURE 3:**
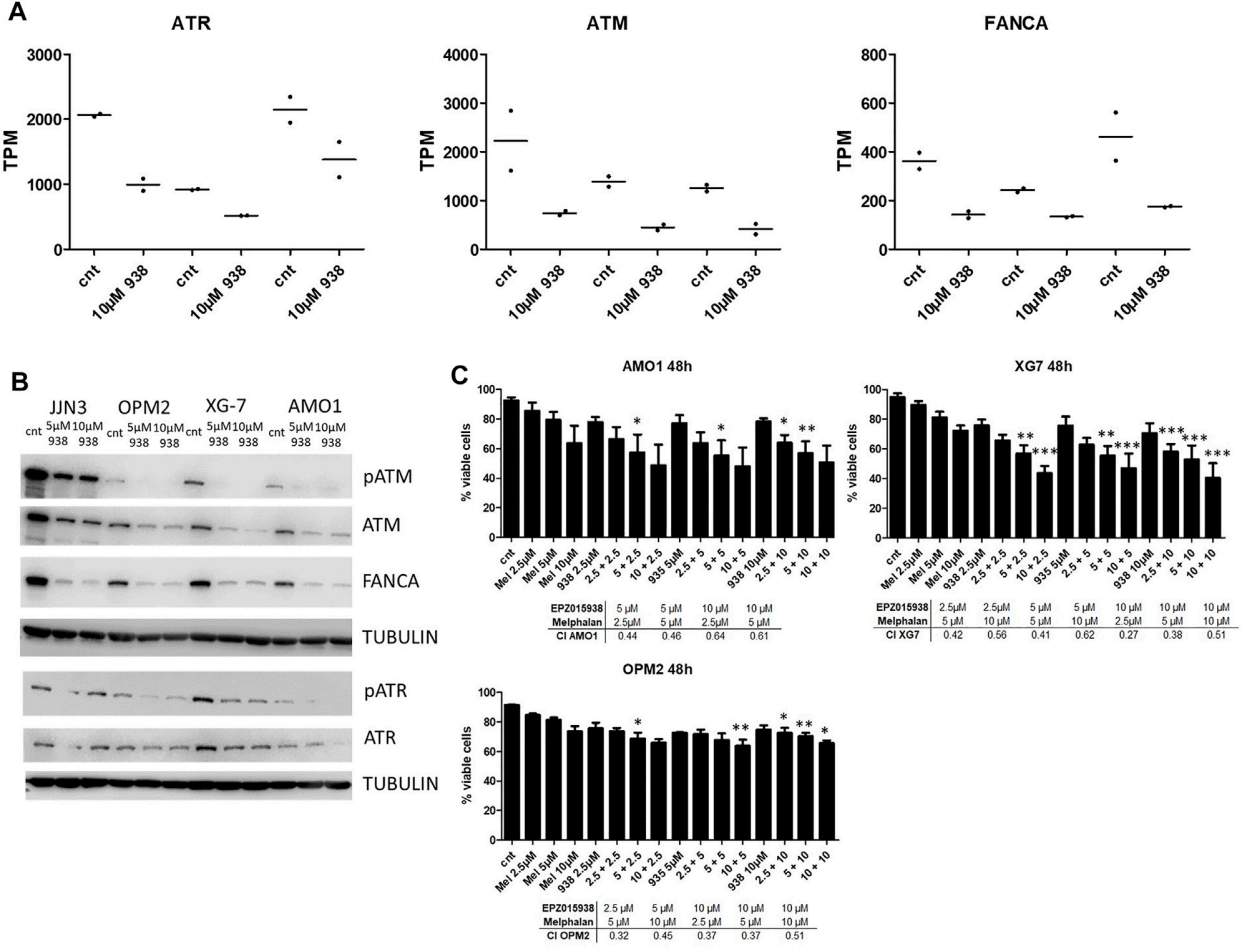
**(A)**: TPM values for ATR, ATM and FANCA for both control and EPZ015938 cells, showing decrease in transcript abundancy following PRMT5 inhibition in all 3 cell lines studied (*n* = 2 for each cell line and condition, no error bars are shown). **(B)**: Western blot analysis was performed on JJN3, OPM2, XG7 and AMO1 cell lysates to evaluate ATM/ATR and FANCA protein levels upon PRMT5 inhibition for 3 consecutive days. Tubulin was used as a loading control. One experiment representative of 3 experiments performed is shown (*n* = 3). **(C)**: Viability assay using Annexin/7AAD-staining of HMCLs after treatment with EPZ015938, melphalan or combination therapy at depicted concentrations. Samples were analysed using an ANOVA selected pairs test. to evaluate differences between different treatment settings and against control samples and samples treated with only EPZ015938 or melphalan. Error bars represent mean +SD; */**/*** denotes *p* < 0.05, *p* < 0.01 and *p* < 0.001 respectively (*n* = 3). Synergy was evaluated for between both compounds by calculation of the combination index (CI) for significant combinations, with a CI < 1 showing a synergistic effect.

To further evaluate the impact of EPZ01598 treatment on DNA repair pathways on a protein level, we performed western blotting for the cell cycle checkpoint kinase Ataxia Telangiectasia Mutated (ATM), the DNA damage sensor ATR Ataxia Telangiectasia And Rad3-Related Protein (ATR) and Fanconi Anemia complementary group A (FANCA) known to play roles in DNA damage repair and shown to be affected by PRMT5 inhibition at the RNA-level (Figure 3A). As such, we saw a decrease of these targets upon PRMT5 inhibition (Figure 3B, Supplementary Figure S7). This result could indicate that HR/FA repair pathways functionality is decreased upon PRMT5 inhibition. In agreement, an increase in gamma-H2AX foci, a biomarker for DNA double strand breaks, was seen upon EPZ015938 treatment (Supplementary Figure S8). Moreover, PRMT5 expression levels were also shown to be significantly correlated with genes involved in HR/FA in MM patients, further strengthening the suggestion that there is cross-talk between PRMT5 function, expression of HR/FA-related genes and DNA damage occurrence (Supplementary Figure S9). We subsequently treated HMCLs with both EPZ015938 and melphalan, an alkylating standard of care agent in MM, to evaluate any potential combinatory effect. We established a sub-lethal dose of melphalan per HMCL prior to combination experiments (data not shown), after which cells were cultured according to methods described above. Using this approach, a statistically significant effect of combination therapy on HMCL survival could be seen in all cell lines used. Cells treated with both compounds had higher levels of cellular death upon AnnexinV/7AAD-staining when compared to control or both compounds in monotherapy. Also, the effect on cellular survival could be seen in HMCLs with a defective p53 state, suggesting the presence of regulatory effects that are irrespective of the genomic status of p53. Calculation of the combination index revealed a synergistic effect between EPZ015938 and melphalan (Figure 3C).

### mTOR Signalling is Important for the Anti-Myeloma Effects of PRMT5 Inhibition in HMCLs

**FIGURE 4:**
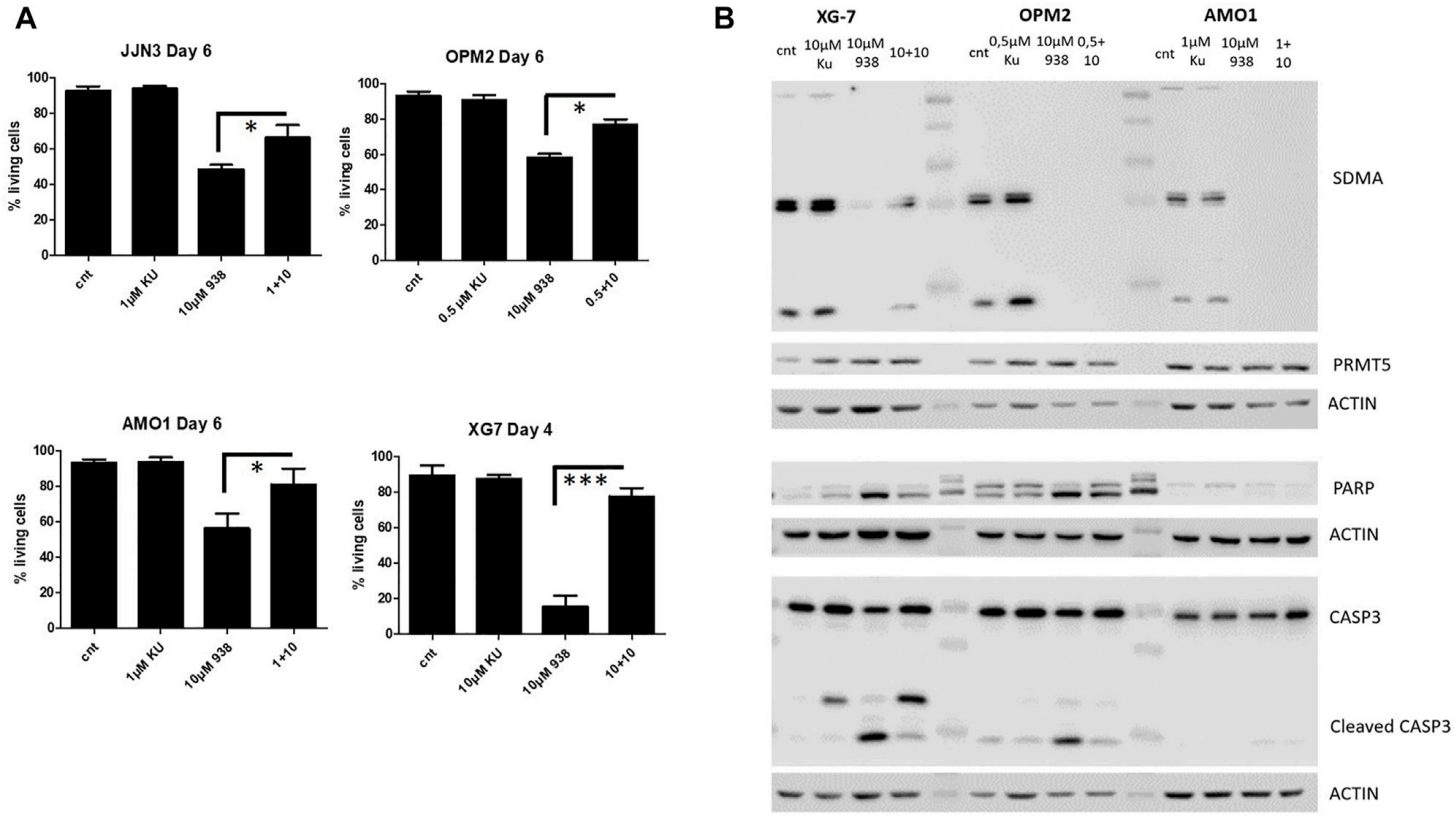
**(A)**: Viability assay of HMCls upon treatment of JJN3, OPM2, AMO1 and XG7 cells with EPZ015938, KU-0063794 or combination therapy after predefined timepoints. Cellular viability was assessed using AnnexinV/7AAD staining. Samples were analysed using an T—test with selected pairs test, significance was evaluated between different treatment settings and compared to control. Error bars depict mean ± SD; */**/*** denotes *p* < 0.05, *p* < 0.01 and *p* < 0.001 respectively (*n* = 3). **(B)**: Western blot analysis of SDMA levels, global PRMT5 levels and pro-apoptotic proteins caspase 9, 3 and PARP in XG7, OPM2 and AMO1 cells. Cells were treated with EPZ015938, KU-0063794 or combination therapy. Control samples were included for comparison. Actin was used as a loading control. One experiment representative of 3 experiments performed is shown (*n* = 3).

A similar approach was used to evaluate whether manipulation of mammalian target of rapamycin (mTOR) signalling, which has been known to play a role in B-cell malignancies, could impact cellular survival when combined with PRMT5 inhibition. We first determined sub-lethal doses of KU-0063794 for each cell line, ranging between 0.5 and 10 µM (data not shown). KU-0063794 reversed EPZ015938 anti-apoptotic effects, leading to diminished cellular toxicity when HMCLs were treated with a combination of both compounds (Figure 4A). We observed a decrease in SDMA levels and decreased caspase cleavage on WB, when cells where exposed to both mTOR- and PRMT5-inhibition. mTOR-inhibition thus leads to the decreased effect of PRMT5 inhibition in HMCL cells (Figure 4B). No significant different increase of PRMT5 on both the RNA or protein levels were seen upon treatment, (Figure 4B; Supplementary Figures S10A,B). Treatment of HMCLs with KU-0063794 alone or in combination with EP2015938 also leads to an increase in AMP-activated protein kinase (AMPK) phosphorylation in XG7 and OPM2 cells whereas monotherapy with EPZ015938 did not, further implicating the interplay between PRMT5 and mTOR/autophagy pathways (Supplementary Figure S11). During exploration of downstream effects, we observed that phosphorylation of 4EBP1 is seen upon treatment with KU-0063794, showing that this compound inhibited mTOR activity in MM cells. Moreover, this observation was also observed in the combination treatment. Effects were less marked in AMO1 cells, potentially due to a more intrinsic resistance (Supplementary Figure S11).

### PRMT5 Inhibition Leads to Defective Splicing in HMCLs and Targets Known MM Pathways

**FIGURE 5:**
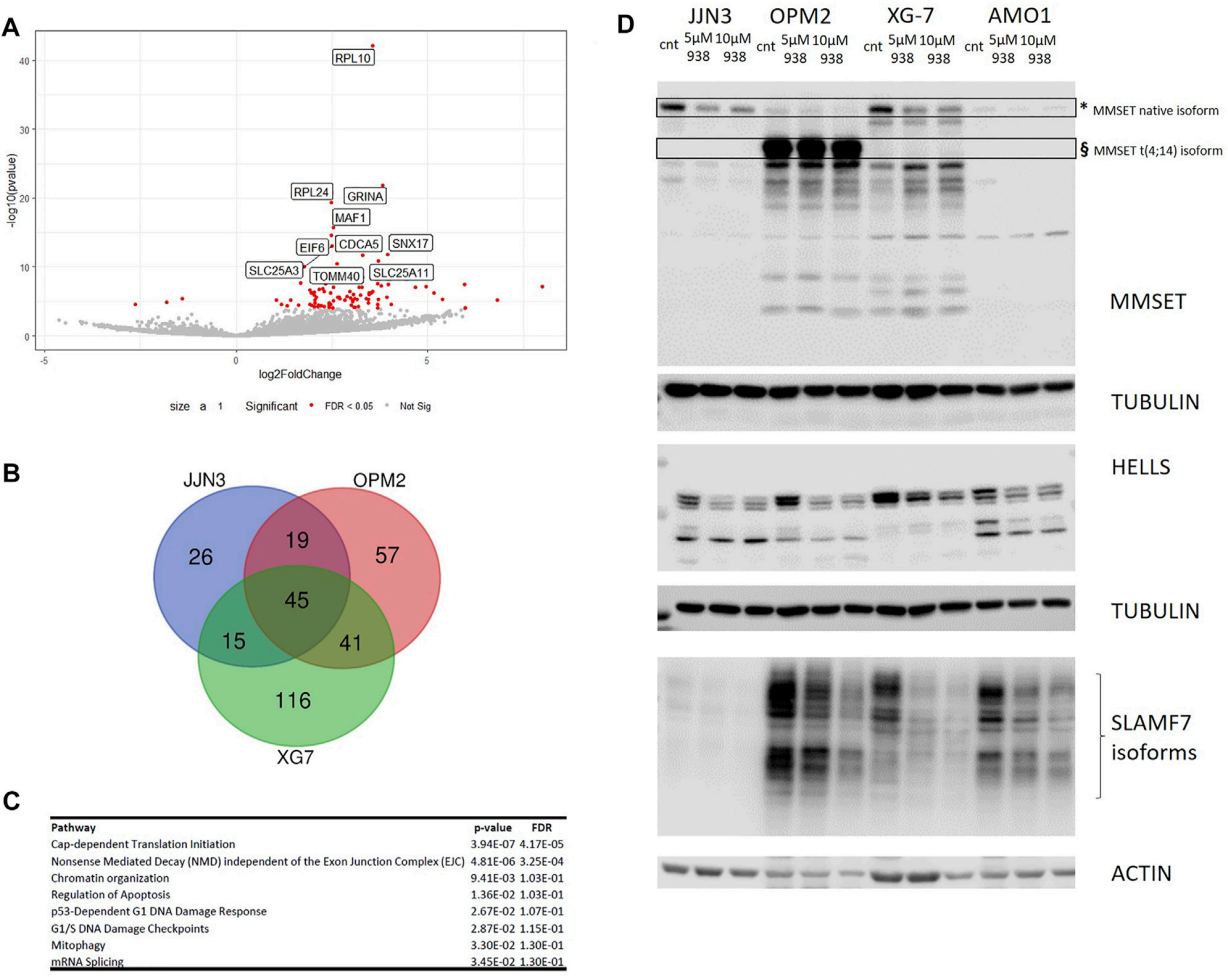
**(A)**: Intron retention due to PRMT5 inhibition was evaluated by re-analysis of RNA-seq data (as described earlier) using IRFinder (*n* = 2 for each condition and per cell line). Volcano plot shows IRFinder output in JJN3 cells, identifying different gene targets affected by intron retention. Only the plot for JJN3 is shown here, other plots are shown in the supplementary data. **(B)**: Venn diagram presentation of common genes affected by intron retention (*n* = 2 × 2 per HMCL/treatment condition). A set of 45 common affected genes were identified. Samples were analysed per cell line type to account for cell type specific effects. **(C)**: Reactome enrichment analysis was subsequently performed using the 45 common genes. Selected pathways that were deregulated are shown. **(D)**: Western blot analysis of identified targets MMSET/WHSC1 HELLS and SLAMF7 in JJN3, OPM2, XG7 and AMO1 cells. For MMSET, * depicts the wild type MMSET isoform where § depicts the aberrant MMSET protein affected by t (4; 14), which is present in OPM2 cells. Tubulin and actin were used as loading control. One experiment representative of 3 experiments performed is shown (*n* = 3).

We next assessed RNA metabolism and splicing. To evaluate the presence of intron retention, RNA-seq output data was re-analysed using IRFinder. Aberrant RNA transcripts could indeed be identified in OPM2, JJN3 and XG-7 cells (Figures 5A–C, Supplementary Figure S12, Supplementary Table S3). When comparing the sets of affected transcripts, a total set of 45 affected genes were shown to be shared between all 3 cell lines studied. Of note, 3 genes of interest were identified as having intron retention in both OPM2 and XG7 cells: 1) HELLS, encoding a lymphoid-specific helicase involved in chromatin remodelling, 2) SLAMF7, encoding the CD319 surface antigen which is targeted by the anti-MM drug elotuzumab and 3) WHSC1/MMSET, involved in the chromosomal translocation t (4; 14). HELLS was also part of our initial high-risk gene signature (Supplementary Table S1). Validation of IRFinder results were performed for HELLS, WHSC1 and SLAMF7. As such, we were able to confirm enrichment for gene transcripts with intron retention for WHSC1 in OPM2 and XG7 cells and for SLAMF7 in OPM2, AMO1 and XG7 cells. A statistical non-significant trend towards upregulation of WHSC1 transcripts was seen in AMO1 cells. Significant intron retention of HELLS gene transcripts was only seen in XG7 cells (Supplementary Figure S13). As intron retention can activate the process of nonsense-mediated decay of RNA transcripts, we evaluated the presence of these proteins, with and without EPZ015938 treatment, showing a decrease in expression levels upon PRMT5 inhibition (Figure 5D). MMSET/WHSC1 levels were less affected in OPM2 cells, which could be due to the presence of the t (4; 14) translocation in these cells. The native WHSC1 isoform was downregulated by PRMT5 inhibition, whereas an additional unaffected protein was observed in OPM2 cells (Figure 5D, * and §, respectively). These findings show that WHSC1 protein levels, in the presence of t (4; 14), are less susceptible to PRMT5 inhibition. This however also suggests a t (4; 14) independent mechanism by which PRMT5 inhibition exerts its function.

## Discussion

**FIGURE 6:**
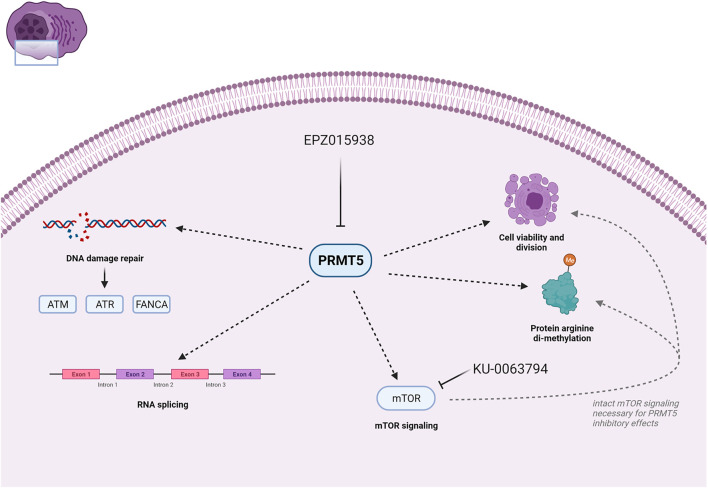
Schematic representation of PRMT5 function and effects upon treatment as evaluated in this study. PRMT5 inhibition by using i.e., EPZ015938 leads to decreased DNA damage repair through a decrease in ATM/ATR and FANCA transcript and protein levels. Moreover, the function of PRMT5 as a regulator of mRNA splicing also plays a role in MM cellular processes and survival. Lastly, our study shows that mTOR signalling is important for PRMT5 function and has effects on cell viability, division and overall arginine di-methylation. Created with BioRender.

In our present study, we were able to confirm that PRMT5 gene expression is associated with adverse PFS and OS in MM patients. Using the PRMT5 inhibitor EPZ015938, we show PRMT5 inhibition to decrease MM cell growth rate, cell count, and increase apoptosis both in HMCLs and in primary human MM samples depicting different of the known molecular defects. Although prior research (Hamard et al., 2018) has identified PRMT5 as being a regulator of p53, we observed that PRMT5 acts in both a p53 dependent mechanism alongside p53 independent mechanisms, as HMCLs harbouring a p53 mutation or del (17p) also displayed increased levels of apoptosis despite the absence of augmented p53 activity. As such, PRMT5 inhibition is of interest in the treatment of MM patients independent of p53 status. Gulla et al. showed that p53 knockdown using shRNA in AMO1 cells could not abrogate the sensitivity to the PRMT5 inhibitor EPZ015666, whereas OPM2 cells, harbouring a gain-of-function mutation in the p53 gene, displayed a resistance to this inhibitor, we saw that EPZ015938 was able to overcome this resistance (Gulla et al., 2018). This difference could be explained by the improved efficacy of EPZ015938 when compared to EPZ015666, as shown by a biochemical IC50 of 6.2 ± 0.8 nmol/L for EPZ0159838 versus 22 ± 14 nmol/L for EPZ015666 (Vinet et al., 2019). Although PRMT5 inhibition has been shown to lead to a G1-arrest in solid cancers such as bladder urothelial cancer and glioblastoma (Banasavadi-Siddegowda et al., 2018; Tan et al., 2020), our data suggests that the mechanism of decreased cellular growth is more complex in MM: CCA profiles could not show G1-arrest in all HMCLS, as thus suggesting the presence of other pathways. NF-κB was already identified as playing a role in PRMT5 inhibition in MM (Gulla et al., 2018). We aimed to further unravel the role of PRMT5 in MM by performing transcriptome analysis. We were able to identify PRMT5 function as being involved in DNA damage repair pathways, mTOR signalling and RNA metabolism. As the alkylator melphalan is a standard of care drug e.g., in autologous stem cell transplantation, we assessed whether melphalan activity could be enhanced by addition of EPZ015938. We found a potentiating effect of PRMT5 inhibition on melphalan treatment, irrespective of p53 status, and potentially caused by modulation of the ATM/ATR and FANCA pathways. As p53 −/− cells have been shown to lose sensitivity to melphalan and other classical chemotherapeutic regimens, this observation creates a rationale for evaluating the role of PRMT5 inhibitors in MM patients harbouring p53 defects (Drach et al., 1998; Munawar et al., 2019). The added benefit of PRMT5-inhibition during treatment with melphalan could as such provide evidence to explore whether pre-treatment of MM patients with a PRMT5 inhibitor prior to exposure to melphalan in a transplant setting would have an added benefit in patients with high-risk MM such as del (17p). Additional evidence for the clinical use of PRMT5 inhibitors has been generated by the results of the METEOR-1 trial, a phase 1 study showing safety and tolerability of the compound in patients with advanced solid tumours (Siu et al., 2019). Moreover, additional clinical trials are currently actively evaluating the clinical use of EPZ015938. Secondly, we observed deregulation of mTOR signalling by PRMT5 inhibition. The mTOR pathway is a critical pathway in cancer cell survival, proliferation and invasion and inhibition of mTOR activity has been shown to lead to decreased MM cell survival (Li et al., 2020). Interplay between mTOR and PRMT5 has already been identified in different cell types, including T-lymphocytes B-cell lymphoma and glioblastoma (Holmes et al., 2019; Webb et al., 2019; Zhu et al., 2019). We observed an opposite phenomenon in MM cells than previously described by Holmes et al. in glioblastoma, an aggressive type of brain malignancy (Holmes et al., 2019). Whereas they saw an increase in PRMT5 function upon mTOR inhibition, our data suggest that PRMT5 activity needs mTOR signalling to exert its function in MM. Secondly, no significant decrease in PRMT5 protein levels was observed upon treatment with an mTOR inhibitor in MM, whereas this was previously reported in T-cell expansion in a murine multiple sclerosis model (Webb et al., 2019). On the contrary, we observed a trend towards upregulation upon mTOR inhibition which is in line with the findings of Holmes et al. in both glioblastoma cell lines and primary patient material. Our observations thus suggest a different relationship between PRMT5 and mTOR signalling, and warrants caution when exploring the options of PRMT5 targeting in clinical practice. Also, the loss of PRMT5 function on EPZ015938 treatment upon mTOR inhibition provides evidence that mTOR pathway modulation could be responsible in part for the observed p53-independent effects on HMCL survival (Yan et al., 2021). A potential explanation in MM specifically could however be the functional effects of cereblon (CRBN) in MM cells and its effects on mTOR signalling. Previous research has shown that deficient CRBN function impacts protein synthesis through the AMPK-mTOR cascade (Lee et al., 2014). Moreover, CRBN is an important target in MM cells and interaction with AMPK has also been seen in HMCLs (Zhu et al., 2011; Zhu et al., 2013). As such, further research towards the interaction between PRMT5 and CRBN would be of special interest, as it is important to evaluate whether PRMT5 inhibition would negate the potential effects of immunomodulatory drugs such as thalidomide and lenalidomide or vice versa (Zhu et al., 2013). A further pathway of interest was the implication of PRMT5 in RNA metabolism and transcript splicing in MM cells. Although PRMT5 has been implicated in spliceosome regulation in other cancers, we provide evidence that intact PRMT5 function is also important for spliceosome function in MM (Rengasamy et al., 2017; Radzisheuskaya et al., 2019). *In silico* analysis was used to identify possible targets of interest of PMRT5 in MM. We saw PRMT5 to be involved in the correct splicing of MMSET/WHSC1, SLAMF7 and HELLS, thus possibly leading to decreased protein levels due to nonsense mediated decay. Subsequent qPCR analysis indeed showed that a significant enrichment of gene transcripts with intron retention were present for SLAMF7 and WHSC1. Aberrant HELLS transcripts were only seen to be enriched in XG7 cells. The presence of a statistically significant downregulation in AMO1 cells upon treatment could also suggest the presence of other mechanisms concerning this gene. 1) MMSET/WHSC1 is aberrantly expressed in MM cells harbouring the translocation t (4; 14) (Keats et al., 2005; Xie and Chng, 2014; Xie et al., 2019). Moreover, it is a high-risk disease marker (Keats et al., 2003; Shah et al., 2016). We observed decreased levels of MMSET protein in HMCLs upon PRMT5 inhibition. In OPM2 cells, a less clear decrease was seen for several isoforms. As these cells harbour the translocation t (4; 14), with marked MMSET overexpression under the IgH promotor in these cells, more discrete alterations at the protein level could however be obscured. 2) SLAMF7, was further assessed due to its implications for possible combination therapy with elotuzumab, a monoclonal antibody targeting SLAMF7 which is already used in MM patient care (Gentile et al., 2021). A decrease in the different isoforms of the SLAMF7 protein could be seen, aside from a marked HMCL specific expression pattern. 3) Lastly, our study identified HELLS, a gene encoding a lymphoid-specific helicase involved in chromatin remodelling, as being processed by PRMT5 during intron removal. It has an important function in B-cell maturation and germline mutations have been shown to cause a severe immunodeficiency syndrome (He et al., 2020). To conclude, we show that the role of PRMT5 in MM disease is much more complex as thought and involves the regulation of DNA damage repair and correct intron removal during gene transcription in MM cells. Moreover, intact mTOR signalling seems to be required for proper PRMT5 inhibitor effects (Figure 6). As such, it is important that PRMT5 is further validated as a potential therapeutic target in a preclinical setting.

## Data Availability

The datasets presented in this study can be found in online repositories. The names of the repository/repositories and accession number(s) can be found below: https://www.ebi.ac.uk/arrayexpress/, E-MTAB-11489 https://www.ebi.ac.uk/arrayexpress/, E-MTAB-372 https://www.ncbi.nlm.nih.gov/geo/, GSE2658 https://www.ncbi.nlm.nih.gov/geo/, GSE19784 https://www.ncbi.nlm.nih.gov/geo/, GSE9782.
