# Supplementary material for: Inhibition of the Protein Arginine Methyltransferase PRMT5 in High-Risk Multiple Myeloma as a Novel Treatment Approach
Source: Front Cell Dev Biol. 2022 Jun 8;10:879057. doi: 10.3389/fcell.2022.879057 (PMC9213887; doi:10.3389/fcell.2022.879057)
Supplement: Supplementary file 3 [file DataSheet1.pdf]

Supplementary Material

1 Supplementary Tables

Supplementary table S1 : List (n = 457) of genes involved in epigenetic and DNA repair pathways used for evaluation in patient cohorts (tab 1); list of prognostic subset of genes in TT2 cohort (tab 2); list of prognostic subset of genes in HM cohort (tab 3); list of common prognostic genes (tab 4); synopsis of clinical data of primary MM patients.

Supplementary table S2 : DESeq2 output for JJN3, OPM2 and XG7 cells (tab 1 to 3); List of significantly deregulated genes per cell line (tab 4); list of common genes between cell lines (tab 5).

Supplementary table S3 : IRFinder output for JJN3, OPM2 and XG7 cells (tab 1 to 3); List of genes with significant intron retention per cell line (tab 4); list of common affected genes between cell lines (tab 5).

2 Supplementary Figures

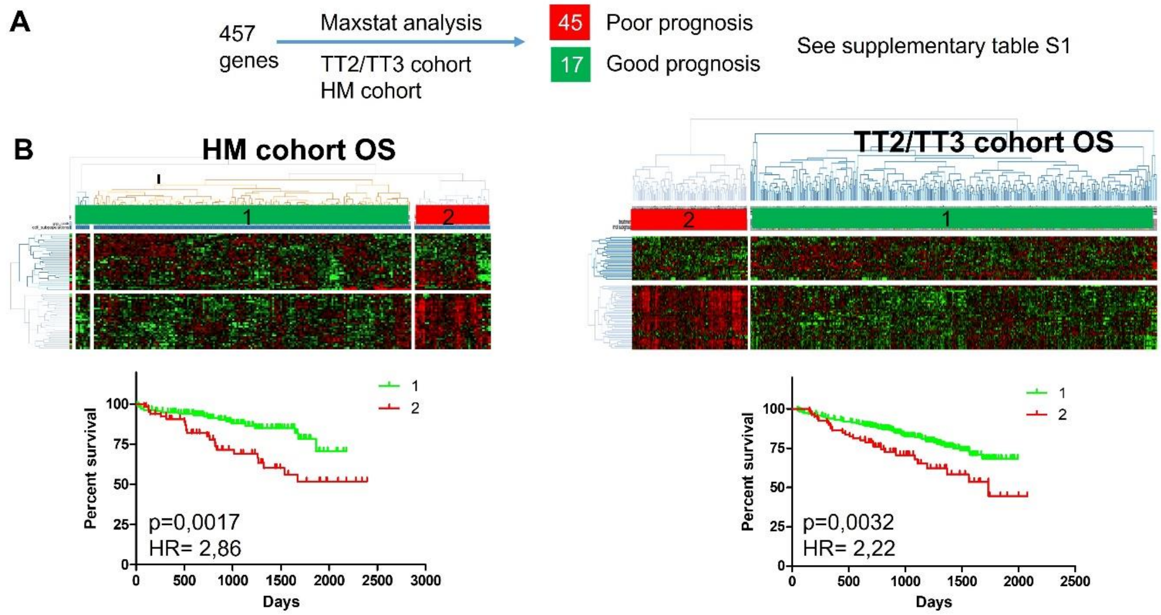

Supplementary Figure S1. A : Bioinformatics workflow for selection of prognostic gene set, starting from 457 selected genes with known involvement in epigenetic and DNA repair pathways. B : Unsupervised hierarchical clustering using 62 prognostic genes in the HM and TT2/TT3 cohort, showing the delineation of a high- and low-risk MM population in both cohorts. In both cohorts a clear identification of a high-risk MM population was possible using the 62 prognostic genes.

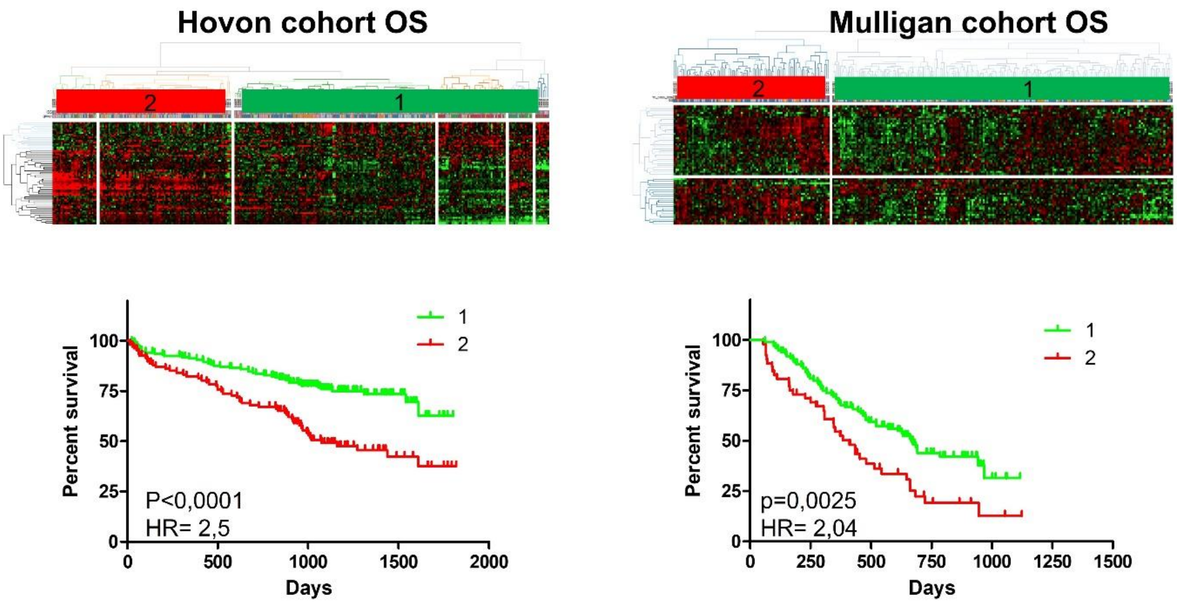

Supplementary Figure S2. Validation of the high-risk gene signature in 2 additional, independent cohorts (GEP/array based) using unsupervised hierarchical clustering, and confirming the validity of the high-risk signature. In both the HOVON and Mulligan cohort, the signature retained the

prognostic value. As the Mulligan cohort consists of heavily pre-treated patients, validity was shown in RRMM as well.

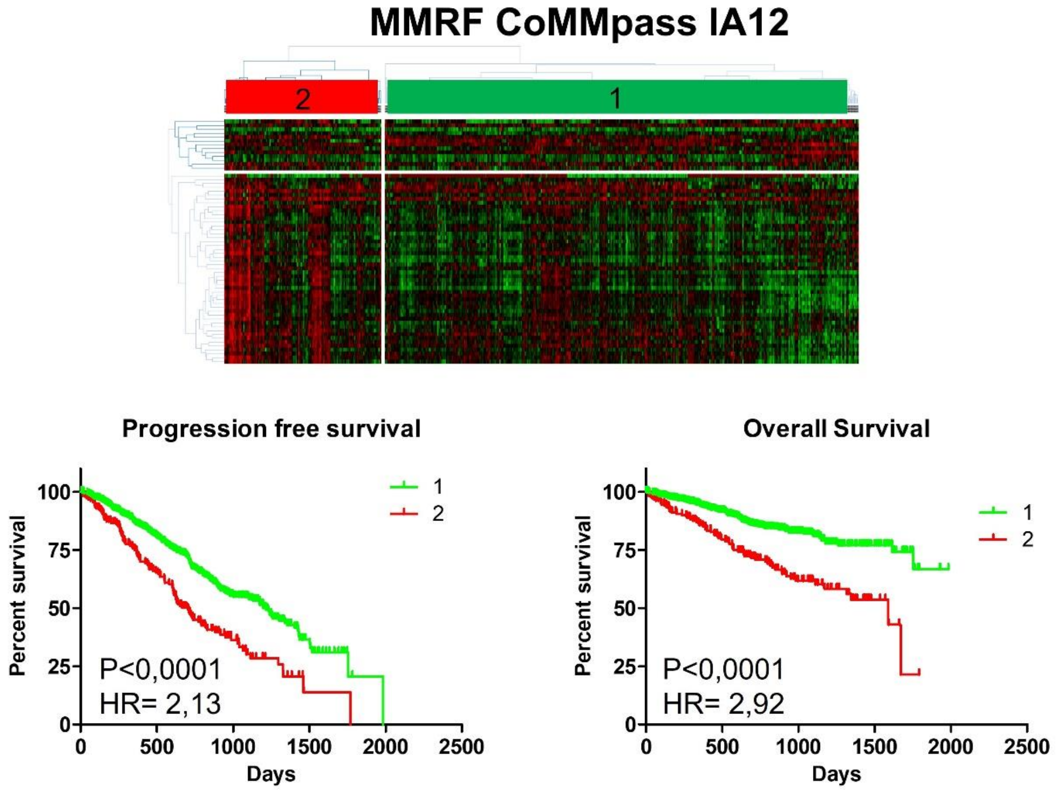

Supplementary Figure S3. Validation of high-risk gene signature in the MMRF cohort (IA12, RNA-seq based) consisting of newly diagnosed MM patients. Unsupervised hierarchical clustering shows discriminatory power both at the level of PFS and OS which show a clinical and statistical difference using Kaplan-Meier/log rank analysis.

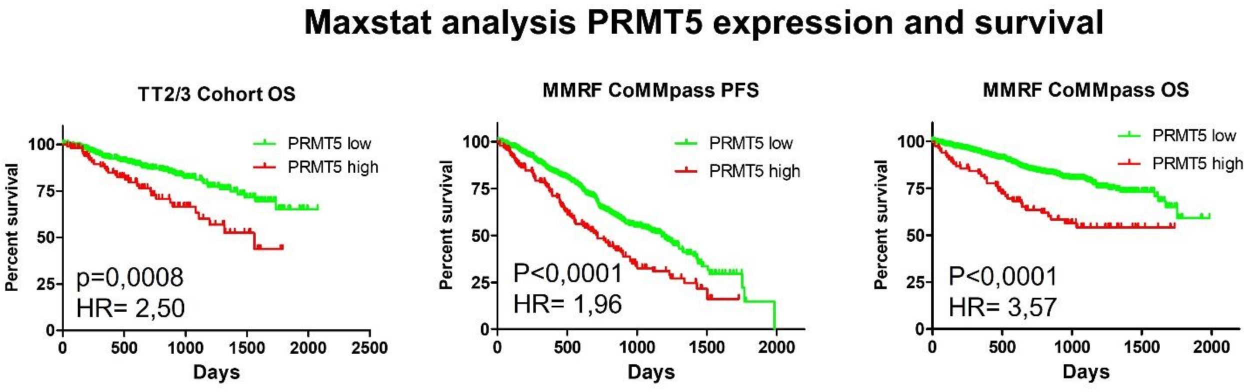

Supplementary Figure S4. Maxstat analysis of candidate gene PRMT5. High expression levels are correlated with a high-risk phenotype in both the TT2/3 and MMRF cohort.

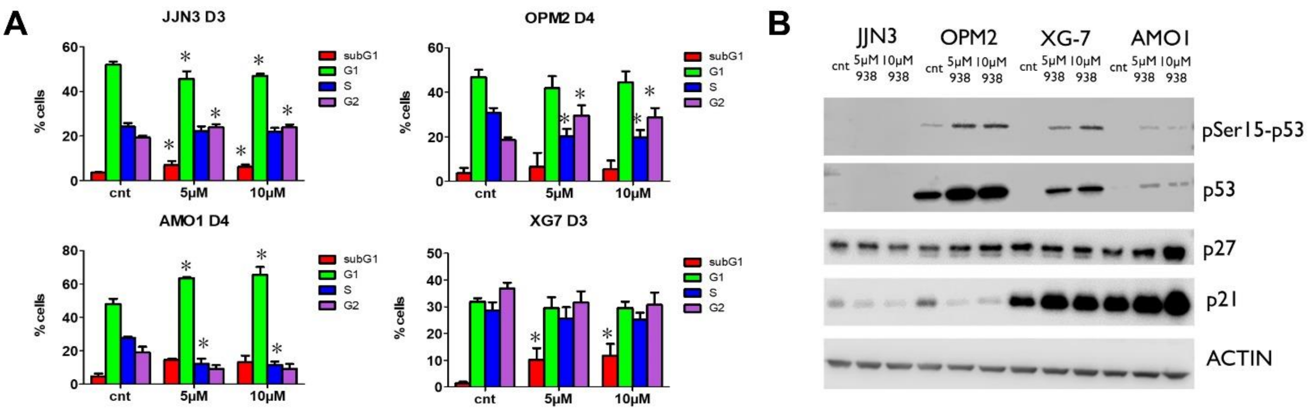

Supplementary Figure S5. A : Cell cycle analysis (n = 3) for each cell line using PI staining. Cell cycle effects were HMCL specific and no clear G1 arrest was seen. Error bars depict mean +/- SD; * denotes p < <0,05. (n = 3). Treated samples were compared to controls. B : Western blot analysis for analysis of p53 response following EPZ015938 treatment in JJN3, OPM2, XG7 and AMO1 cells. Analysis was performed for p53, phospho-Ser15-p52, p27 and p21. One representative experiment of three performed experiments is shown. (n = 3)

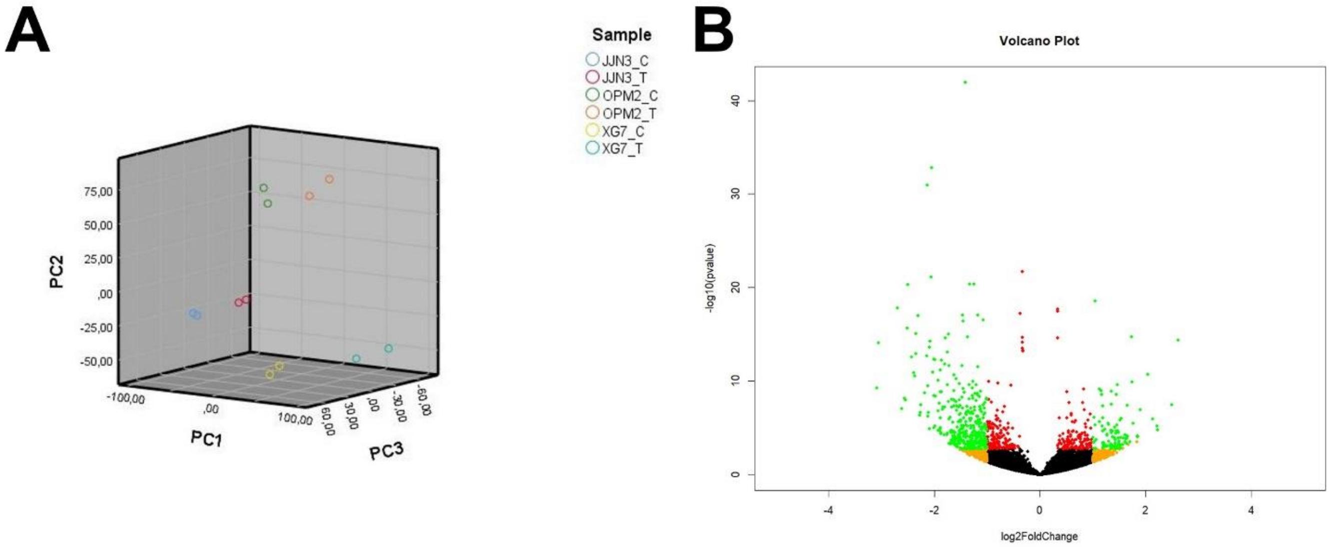

Supplementary Figure S6. A : Principal component analysis plot according to top 3 components of RNAseq data. Plot suggests good reproducibility in replicates but with large influences according to cell line type and treatment. B : Volcano plot depicting analysis and identification of differential gene expression according to Log2(foldchange) and –log10(p-value). Selected genes are depicted in green. One representative plot is shown.

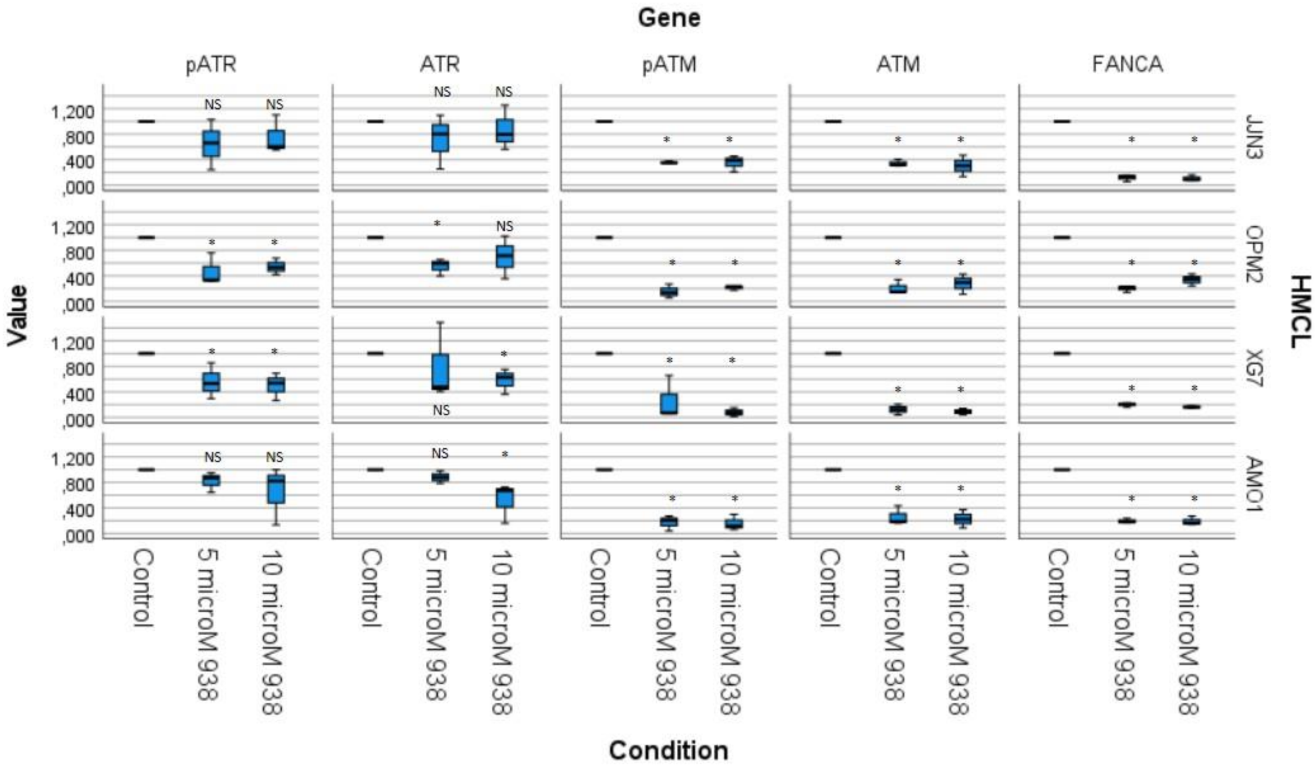

Supplementary Figure S7. Statistical analysis of densitometric values (calculated as optic value of involved gene bands divided by tubulin bands) of western blot data shown in figure 3B. Values were calculated through analysis of raw data images in Image Studio (Li-Cor). Error bars depict mean +/- SD. * denotes $p < 0.05$.; NS denotes not significant. Treated samples were compared against controls.

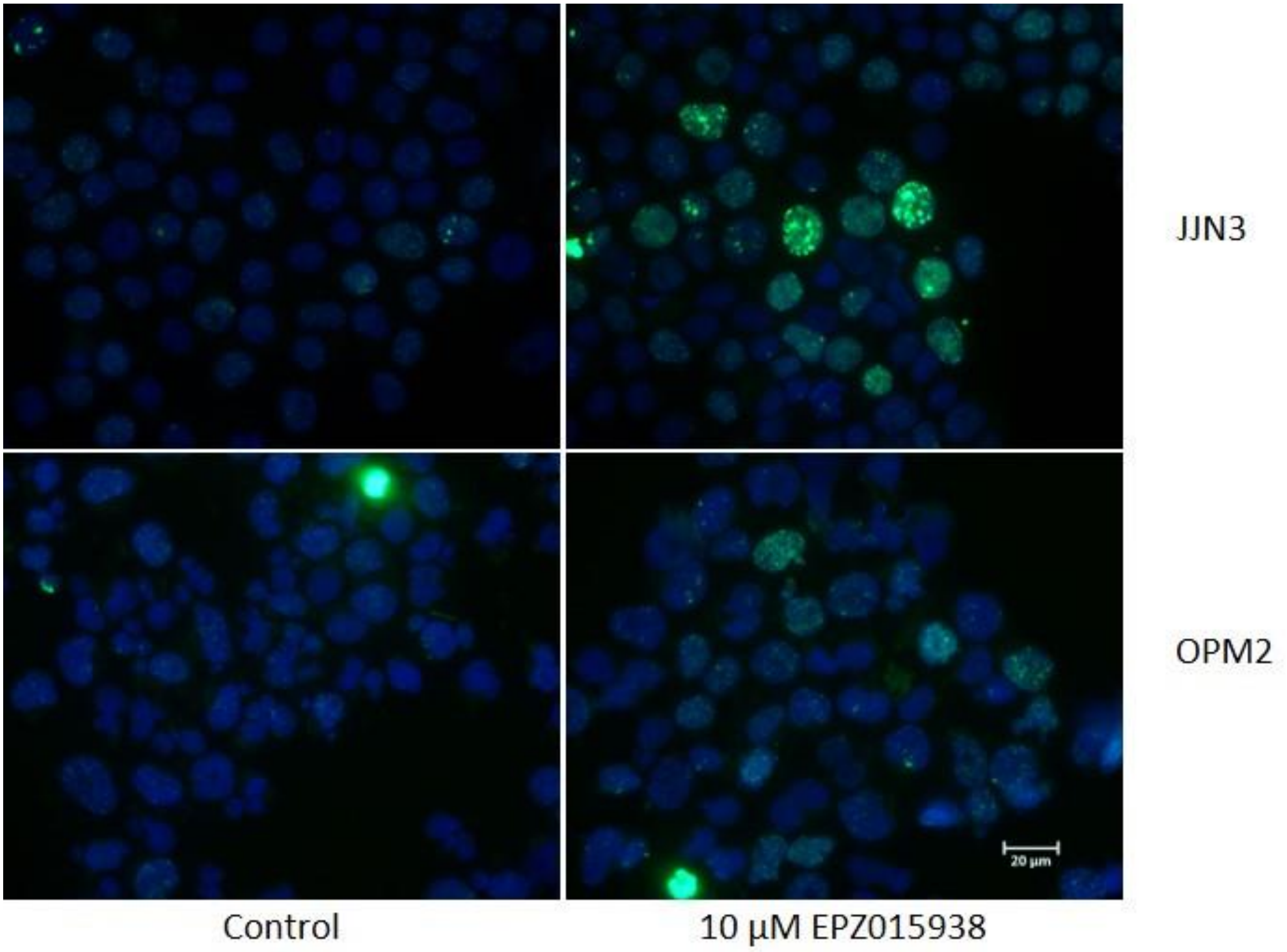

Supplementary Figure S8. Immunofluorescent staining for gamma-H2AX foci in JJN3 and OPM2 cells following three days of 10 µM EPZ015938 treatment versus control. One representative experiment of two performed experiments is shown.

Correlation coefficient	Interpretation	p-value	Gene
,261	Low	< 0,001	GEN1
,271	Low	< 0,001	BARD1
,296	Low	< 0,001	PDS5B
,308	Low	< 0,001	SLX1B
,344	Low	< 0,001	DMC1
,347	Low	< 0,001	NBN
,427	Moderate	< 0,001	SHLD3
,459	Moderate	< 0,001	SLX1A
,460	Moderate	< 0,001	RAD51
,462	Moderate	< 0,001	SMC5
,470	Moderate	< 0,001	EME1
,478	Moderate	< 0,001	RAD54L
,488	Moderate	< 0,001	RAD54B
,502	Moderate	< 0,001	XRCC2
,513	Moderate	< 0,001	RAD51B
,519	Moderate	< 0,001	EME2
,522	Moderate	< 0,001	ABRAXAS1
,534	Moderate	< 0,001	SHLD1
,537	Moderate	< 0,001	HELQ
,538	Moderate	< 0,001	SHLD2
,559	Moderate	< 0,001	SWI5
,564	Moderate	< 0,001	PAXIP1
,568	Moderate	< 0,001	XRCC3
,578	Moderate	< 0,001	BRCA1
,583	Moderate	< 0,001	SEM1
,587	Moderate	< 0,001	SMC6
,591	Moderate	< 0,001	RAD52
,592	Moderate	< 0,001	ZSWIM7
,607	High	< 0,001	SPIDR
,607	High	< 0,001	MUS81
,615	High	< 0,001	RBBP8
,635	High	< 0,001	RAD51D
,665	High	< 0,001	SWSAP1
,683	High	< 0,001	RAD50

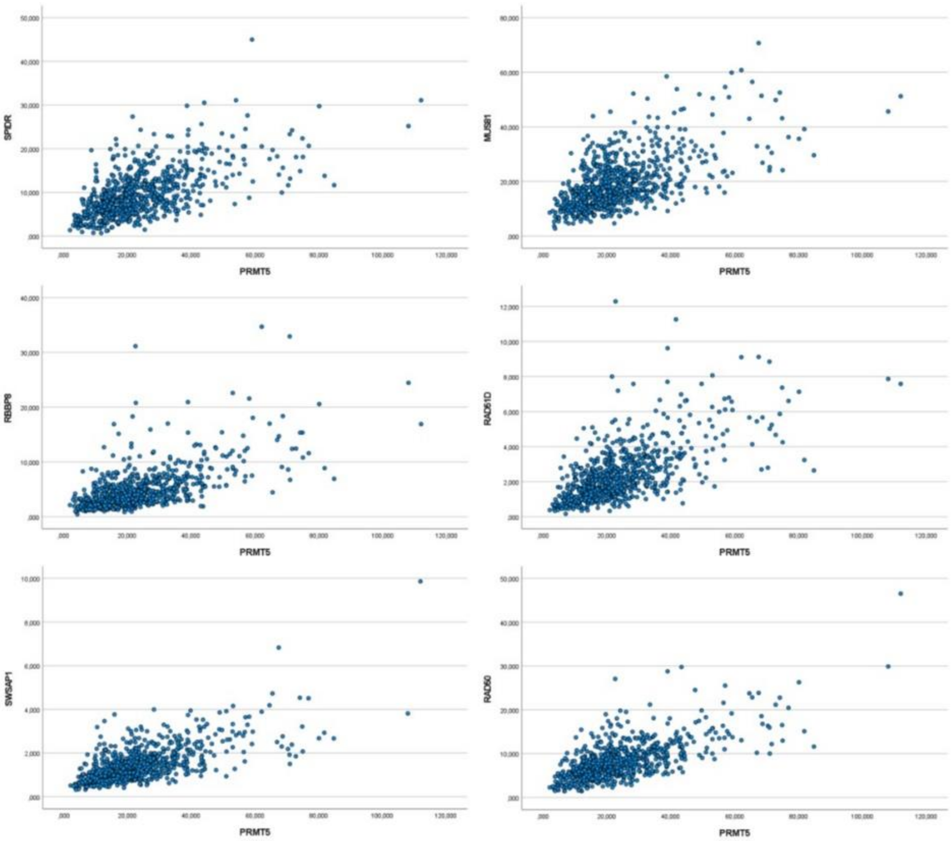

Supplementary Figure S9. Correlation coefficients between expression levels of PRMT5 and genes involved in HR/FA pathways in primary MM patients included in the MMRF cohort (n = 707, available for analysis). Correlation dot plots shown for 6 genes with Pearson coefficient > 0.60.

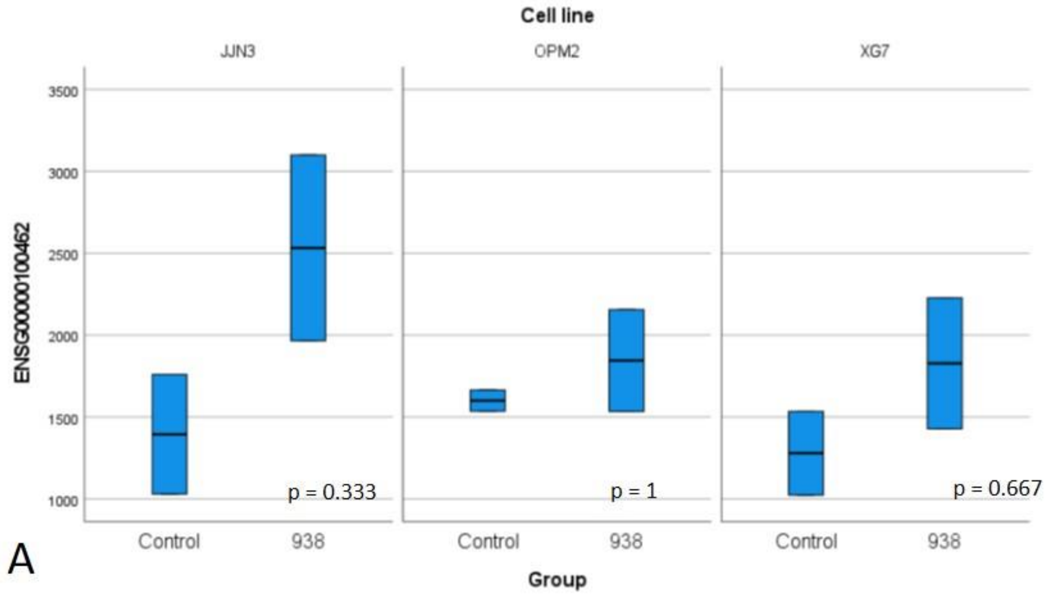

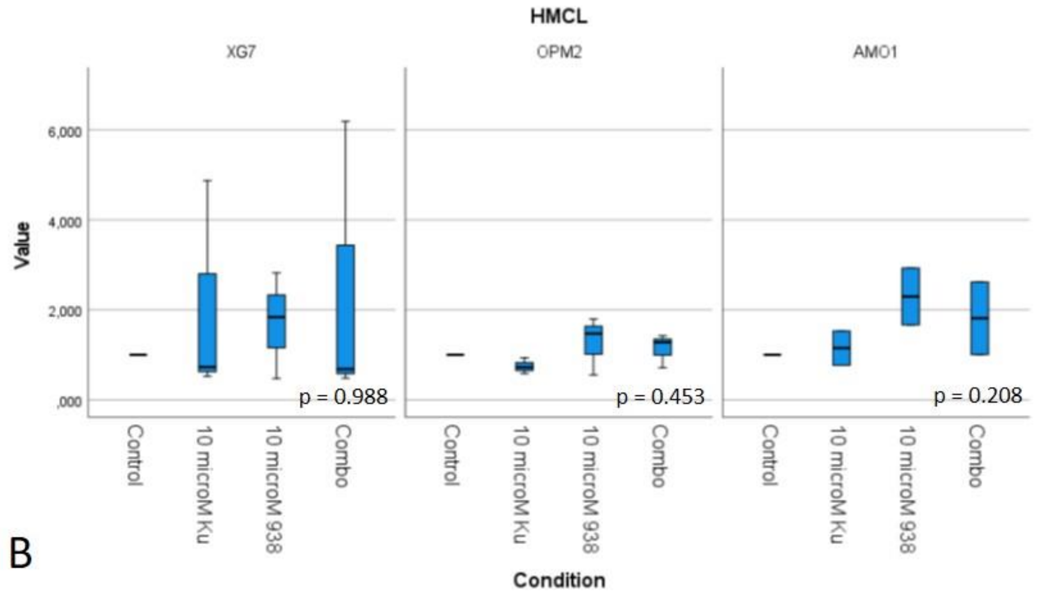

Supplementary Figure S10. A : Statistical analysis of RNAseq data (TPM counts) for PRMT5 (ENSG00000100462) at baseline and after EPZ015938 treatment. Differences in expression levels were not significant. B : Densitometric values (calculated as optic value of PRMT5 bands divided by actin bands) of western blot data shown in figure 4. Values were calculated through analysis of raw data images in Image Studio (Li-Cor). Differences observed were not significant. Error bars depict mean +/- SD

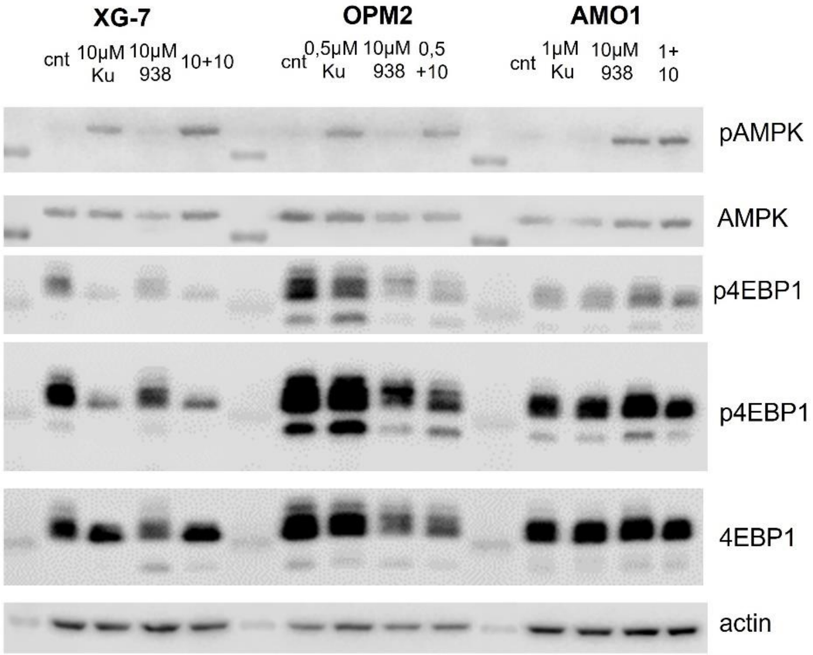

Supplementary Figure S11. Western blot analysis for analysis of mTOR signaling following EPZ015938 and KU-0063794 treatment in OPM2, XG7 and AMO1 cells. Analysis was performed for pAMPK, AMPK, p4EBP1 and 4EBP1. One representative experiment of three performed experiments is shown. Actin was added as a loading control. (n = 3)

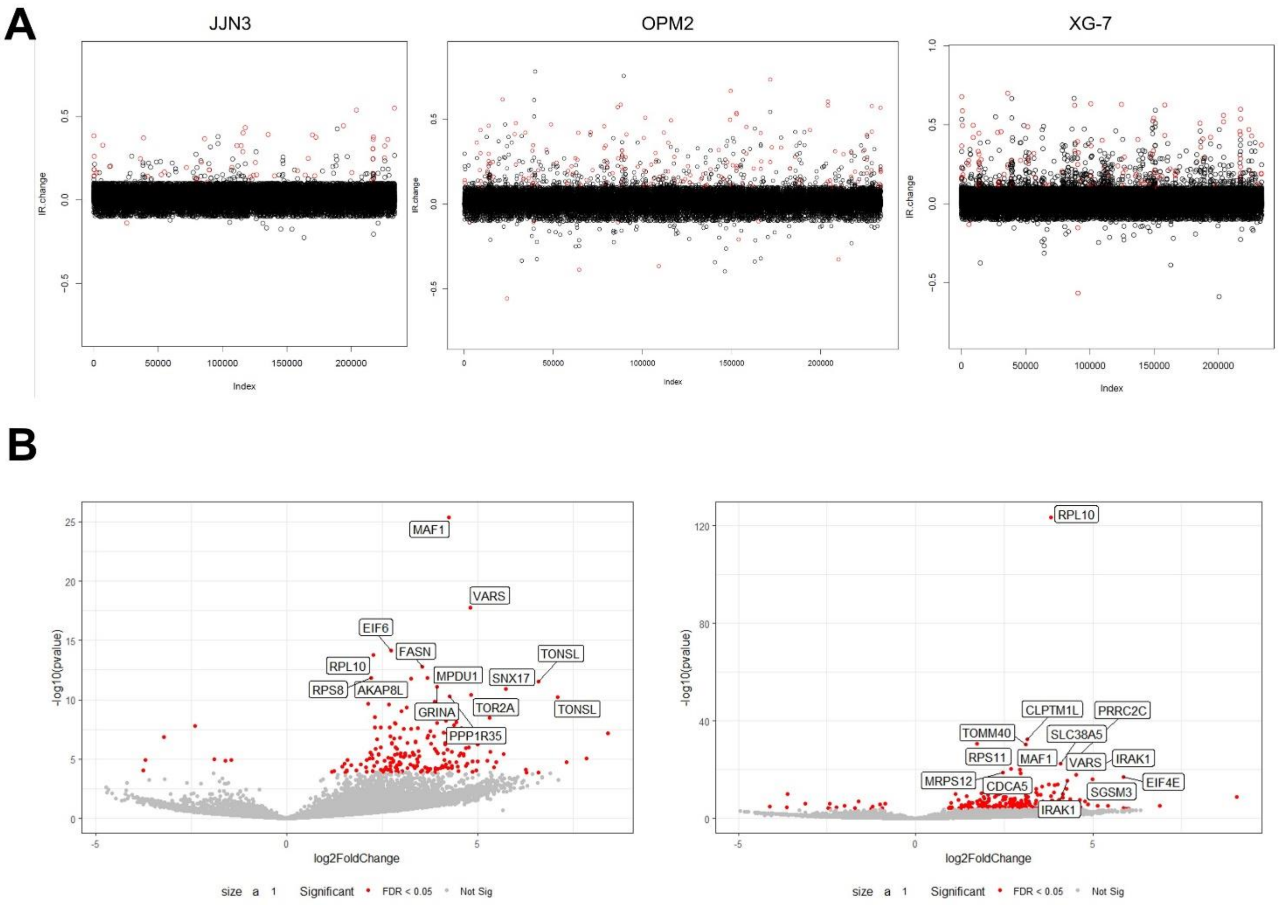

Supplementary Figure S12. A : IRFinder output plots for JJN3, OPM2 and XG-7 cells, showing presence of transcripts with intron retention crossing significance threshold (red dots indicate transcripts enriched with p < 0,05). B : Volcano plot of IRFinder output for XG-7 and OPM2 cells – treated versus untreated conditions (n = 2 for each condition).

A

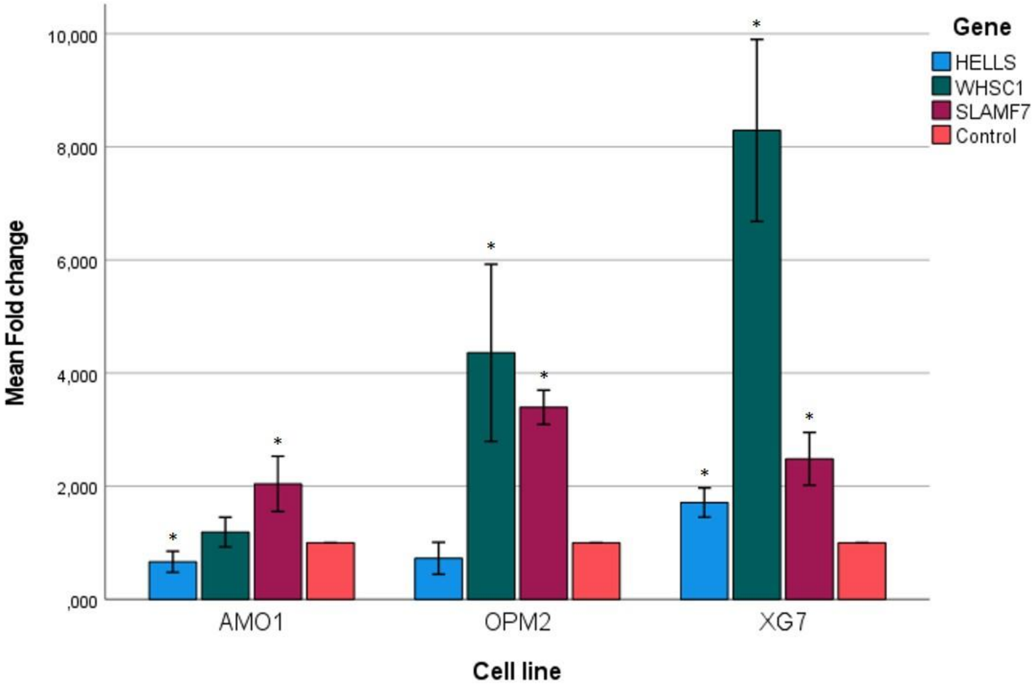

B

Gene	Forward primer (5’-3’)	Reverse primer (5’-3’)
HELLS	AAGGACTTTCCAGTGTTGTTAT	TCTCATCTTTGGGTGTGATTT
WHSC1	CAGTACCCGCTCCAAGAAAT	GATATTAGGGTCGTGAAACCCT
SLAMF7	GAAGCTCTGTGAAGGTGACTG	GGATGTGGAAGAAGTCGAGGAT

Supplementary Figure S13. A : qPCR validation of HELLS, WHSC1 and SLAMF7 transcripts with intron retention as predicted by the IRFinder algorithm. Cells were treated for 3 days with either placebo or EPZ015938. Samples were analysed using a Mann-Whitney U test comparing treated samples versus untreated controls. Error bars depict mean +/-SE, * denotes p<0,05. (n = 3) B : Primer sequences used for this analysis. Primers were designed to target the retained intronic region as predicted by IRFinder : WHSC1 chr4: 1976679- 1978637 / SLAMF7 chr1: 160750423-160751344 / HELLS chr10: 94574187- 94574553.
